# Extremophilic Microfactories: Applications in Metal and Radionuclide Bioremediation

**DOI:** 10.3389/fmicb.2018.01191

**Published:** 2018-06-01

**Authors:** Catarina R. Marques

**Affiliations:** Departamento de Biologia and Centro de Estudos do Ambiente e do Mar, Universidade de Aveiro, Aveiro, Portugal

**Keywords:** extremophilic bacteria and archaea, meta-‘omics’, genetic engineering, synthetic biology, mine wastes, metal-radionuclide recycling/recovering

## Abstract

Metals and radionuclides (M&Rs) are a worldwide concern claiming for resilient, efficient, and sustainable clean-up measures aligned with environmental protection goals and global change constraints. The unique defense mechanisms of extremophilic bacteria and archaea have been proving usefulness towards M&Rs bioremediation. Hence, extremophiles can be viewed as microfactories capable of providing specific and controlled services (i.e., genetic/metabolic mechanisms) and/or products (e.g., biomolecules) for that purpose. However, the natural physiological plasticity of such extremophilic microfactories can be further explored to nourish different hallmarks of M&R bioremediation, which are scantly approached in the literature and were never integrated. Therefore, this review not only briefly describes major valuable extremophilic pathways for M&R bioremediation, as it highlights the advances, challenges and gaps from the interplay of ‘omics’ and biological engineering to improve extremophilic microfactories performance for M&R clean-up. Microfactories’ potentialities are also envisaged to close the M&R bioremediation processes and shift the classical idea of never ‘getting rid’ of M&Rs into making them ‘the belle of the ball’ through bio-recycling and bio-recovering techniques.

## Introduction

Metals and radionuclides (M&Rs) are problematic pollutants sourced from different industrial sectors, nuclear power plants, electronic waste and, especially, from mining activities. Undoubtedly, mining industry nurtures a plethora of human needs (Carvalho, 2017), but past and ongoing mining activities represent a huge footprint of environmental (Marques et al., 2014) and health injuries (Pereira et al., 2014; Venkateswarlu et al., 2016). Still, future societal demands will force industrial growth, thus making M&R pollution a never-ending threat, mainly due to their persistence and non-biodegradability (Gupta and Diwan, 2017).

**FIGURE 1:**
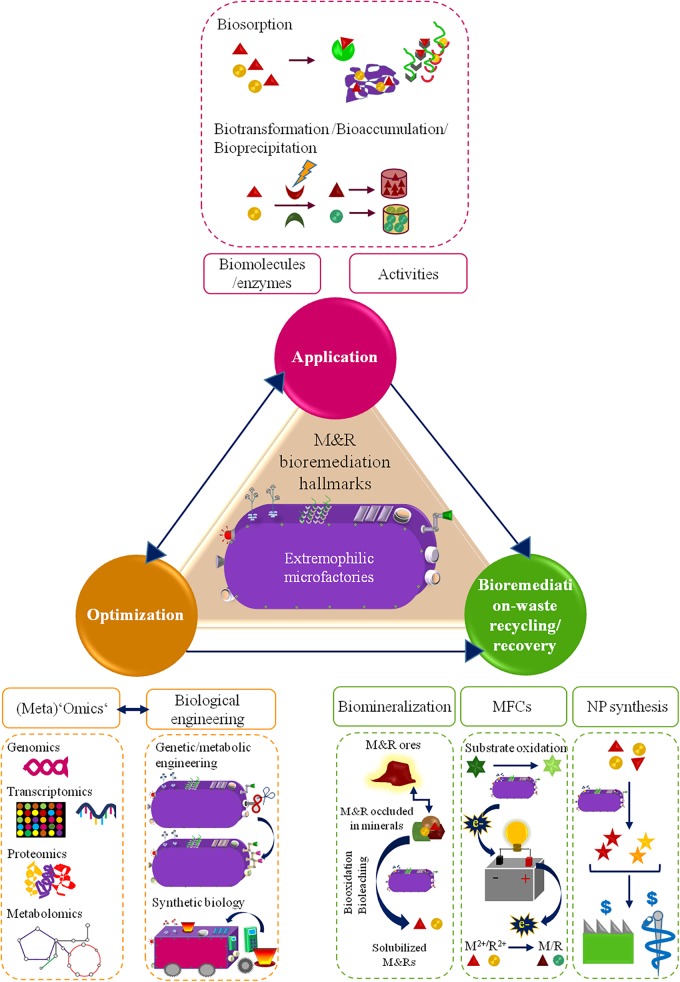
Schematic diagram of the M&R bioremediation hallmarks approached in this review.

Bioremediation, though being often viewed as an ‘old fashion’ and slow-acting technology, it has been thriving for M&R reclamation. Its sustainability, low-cost and efficiency totally rival conventional remediation techniques (Li and Zhang, 2012). In particular, microbially-based bioremediation relies on microbes abilities, biogenic products and/or components to scavange, transform or immobilize M&Rs (Lloyd and Lovley, 2001; Voica et al., 2016). Despite the interesting nature-based self-healing character of this approach, the tolerance of microbes to high M&R levels, extreme physical (e.g., radiation), chemical (e.g., acidic pH) and climate changing conditions may constrain (*in situ*) bioremediation efficiencies (Ramos et al., 2011). Extremophilic microbes, though, have attractive skills as bioremediation tiny factories (microfactories), which performance can even be improved and customized for M&R clean-up. This review will hence focus: (1) the peculiar mechanisms of extremophilic bacteria and archaea exploitable as ‘microfactories’ for M&R bioremediation, (2) the groundbreaking opportunities leveraged from nature and science (‘omics’ plus biological engineering) interplay to enhance extremophilic-microfactories-based bioremediation, (3) M&R recovery and recycling conducted by extremophilic microfactories as a sustainable and value-added management of M&R-bioremediation-resulting wastes. Overall, this is an innovative overview on the potentialities of extremophilic bacteria/archaea in three M&R bioremediation hallmarks: application, optimization, end-waste management (**Figure 1**).

## Extremophiles as Microfactories for M&R Bioremediation

**FIGURE 2:**
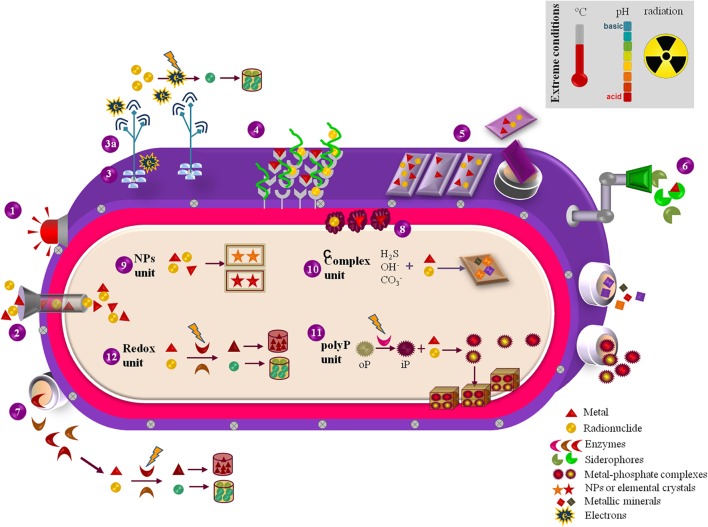
Representation of the exploitable biosynthetic and metabolic features of extremophilic microfactories with relevance for M&R bioremediation purposes. (1) M&R sensing by regulatory proteins that trigger downstream genetic and metabolic pathways coping with M&R exposure. (2) M&R uptake through energy-dependant transporters as resistance nodulation cell division carriers. (3) Surface *c*-cytochromes that promote electron transference to pili (3a), which electron-conductive ability promotes the extracellular reduction and precipitation of M&Rs. (4) M&R binding sites at the cell-surface cationic and anionic functional groups and S-layer proteins. (5) Extracellular polymeric substances (EPS) that function as panels for M&R capture and sorption, either attached to the cell surface or released to the extracellular environment. (6) Biosynthetic siderophores extracellularly excreted for M&R sorption. (7) Redox enzymes liberated for catalyzing the reduction/oxidation of M&Rs that, if reduced, may precipitate and accumulate in the outer environment. (8) Metallic chaperones located in the periplasm space that promote M&Rs quenching and mobilization. (9) M&R-based nanoparticles (NPs) or nanocrystals formed and accumulated in the cytoplasm. (10) M&R biotransformation through complexation with hydroxides, carbonates, sulfides, hence forming M&R-oxides, M&R-carbonates and M&R-sulfides complexes either intracellularly accumulated or released to the exterior environment. (11) Enzymatic hydrolysis of organic poly-phosphates into inorganic phosphates for M&R precipitation and accumulation as metal-phosphate granules in the inner space or expelled to the exterior. (12) Reduction/oxidation of M&Rs in the cytoplasm driven by biosynthesized redox enzymes.

Extremophilic bacteria and archaea evolved specialized mechanisms to endure physical and/or geochemical extreme environments (Rothschild and Mancinelli, 2001). Acidophilic and/or metalophilic microorganisms are especially appealing agents for bioremediation, given their defense mechanisms against M&Rs and acidity. They synthesize extremophilic enzymes (extremozymes) (Elleuche et al., 2014) and biomolecules (Raddadi et al., 2015) that keep active and/or stable under harsh conditions. The stability of thermophilic enzymes, like the esterase EstATII (Mohamed et al., 2013), helps facing acidity and metal stress, thanks to stiff folds sustained by ion-pair networks, to compact hydrophobic cores and aminoacid arrangements/packing (Elleuche et al., 2014). Additionally, extremophiles evolved sharpened metal detoxification pathways (Voica et al., 2016) due to fast-adapting transcriptional and translational mechanisms that activate and/or inhibit many anti-oxidative stress, metal-binding, metal-transport, and membrane-permeability responses (Mukherjee et al., 2012; Dekker et al., 2016). Their membranes exhibit a specific structure (Singh and Singh, 2017), composition and a positively-charged inner layer that promote metal-transporters functioning (Dekker et al., 2016) and minimize metals and protons entrance, thereby controlling acidity and M&R toxicity (Slonczewski et al., 2009; Krulwich et al., 2011; Navarro et al., 2013; Zhang et al., 2016). Notwithstanding, since extremophiles can express defense mechanisms active against multiple extremes simultaneously (Rothschild and Mancinelli, 2001), thermophilic, halophilic, radiophilic, and polyextremophilic bacteria/archaea are additional M&R clean-up agents (Amoozegar et al., 2012; Wheaton et al., 2015). Either as whole-cells or providers of economically-valuable bio-services and/or biomolecules (**Figure 2**), extremophiles can be explored as tiny factories (microfactories) for M&R remediation.

### M&R Sensing

Sensing M&Rs presence is the alarm button to trigger the activation/repression of specific regulatory proteins and downstream cascades that control the expression of resistance genes/operons encoding enzymes and biomolecules for M&R detoxification (Das et al., 2016). Along this, the uptake/efflux of M&Rs across extremophiles cell wall and membranes may occur through energy-dependent P-type ATPases (*copA1_Af_*, *copA2_Af_*, and *copB_Af_*), resistance nodulation cell division carriers (*cusA_Af_*, *cusB_Af_*, and *cusC_Af_*) and/or chaperones (*cusF_Af_* and *copC_Af_*) as in the copper-resistant *Acidithiobacillus ferrooxidans* (Navarro et al., 2009). A comprehensive description of the genetic characteristics and functioning of regulatory and M&R uptake/efflux systems was performed namely for thermoacidophilic, acidophilic, and metalophilic bacteria and/or archaea (Nies, 2000; Orell et al., 2010, 2013; Navarro et al., 2013; Wheaton et al., 2015).

### Biomolecules for M&R Biosorption

Extremophiles-derived cell wall/capsule, S-layer proteins, extracellular polymer substances (EPS) and siderophores present unusual structural and functional properties for M&Rs biosorption under severe stress. Llorens et al. (2012) suggested uranium sorption by S-layer proteins on the metalophilic *Cupriavidus metallidurans* at pH1; while acidophilic (Masaki et al., 2015) and moderate halophilic bacteria/archaea adsorbed M&Rs onto cell surface (Amoozegar et al., 2007, 2008, 2012). EPS are the most relevant structures for M&R biosorption with proved efficiency namely on psychrotolerant (*Pseudoalteromonas* sp.; Qin et al., 2007) and acidophilic (*A. ferroxidans*; Sand and Gehrke, 2006) bacteria. Either attached on the cell surface or extracellularly released, extremophilic EPS promote cell adhesion, biofilm formation, and M&Rs biosorption (Poli et al., 2011) due to a greater abundance of negatively-charged metal-binding sites of its components (e.g., polysaccharides, proteins, nucleic acids, and lipids) (Nies, 2000; Navarro et al., 2013; Shukla et al., 2017). An improved structure/composition favors enzyme stabilization and protein anchoring, hence boosting bacteria/archaea proliferation (Qin et al., 2007). Some halophilic archae can synthesize EPS with specialized jellifying properties due to abundant glucuronic acids and sulfates that stabilize EPS matrix and enhances M&R sorption (Singh and Singh, 2017). Siderophores are small biomolecules released to the environment for metal scavaging. De Serrano et al. (2016) reviewed regulatory mechanisms and chemical features of siderophores synthesized by extremophiles, evidencing their potential as bioremediation agents for metal chelation.

### Biocatalysts for M&R Biotransformation and Bioprecipitation/Bioaccumulation

Extremophiles mediate the chemical transformation of M&R through the biosynthesis of enzymes that intracellularly or extracellularly catalyze M&Rs oxidation (solubilization) or reduction (precipitation) (Das et al., 2016). Overall, the redox reactions may occur through direct or indirect activity of extremophiles (e.g., sulfur-/iron-oxidizing or sulfate-reducing), under aerobiosis and/or anaerobiosis (Zhang et al., 2016; Shukla et al., 2017). Normally, M&R reduction leads to the bioprecipitation/bioaccumulation of organic- and mineral-M&R complexes, and M&R-based nanocrystals. Halophilic (Amoozegar et al., 2012; Srivastava et al., 2013), thermo-tolerant (Özdemir et al., 2017a,b), thermophilic (Kashefi and Lovley, 2000), metalophilic (Lovley et al., 1991), radiophilic (Appukuttan et al., 2011) and acidophilic bacteria and archaea (Orell et al., 2012; Masaki et al., 2015), proved to precipitate tellurite, uranium, iron, silver, chromium, or copper as elemental nanocrystals, carbonate complexes, magnetite and metal-sulfides. Inorganic polyphosphate granules enzymatically-transformed by acidophilic and metalophilic bacteria (e.g., *Caulobacter crescentus*, *Acidithiobacillus* sp.) and archaea (e.g., *Sulfolobus metallicus*) can intracellularly and extracellularly accumulate M&Rs (Hu et al., 2005; Navarro et al., 2009; Orell et al., 2012). M&R bioaccumulation/bioprecipitation was indeed favored by highly reductive pili (Cologgi et al., 2011) and *c*-cytochromes (Marshall et al., 2006; Shelobolina et al., 2007) that increase electron transport, which attached to *Geobacter* sp. or *Shewanella oneidensis* outer membranes, promote the extracellular reduction of U(VI) into the precipitable U(IV) along with cell viability and growth. These strategies are excellent towards *in situ* bioremediation, as already applied for uranium clean-up in groundwater (Coates and Anderson, 2000).

Despite the great biotechnological potential of extremophilic microfactories, remediation efficiency, biomass productivity, and economic profitability may be challenging, especially for *in situ* applications due to extreme optima, interspecies competition and interaction/communication inhibiting bioremediation, and limited proliferation (Ramos et al., 2011; Johnson et al., 2015). Consequently, extremophiles-based M&R bioremediation may become less attractive considering mining industry demands, hence requiring function-directed improvements.

## Upgrading Extremophilic Microfactories for Enhanced M&R Bioremediation

‘Omics’ and biological engineering are leading the achievement of more proficient extremophilic microfactories for M&R bioremediation to keep pace with current economical, technological, social, and climate change defiance.

### ‘Omics’ Package: Unraveling New Genes and Functions of Extremophiles

A fundamental step is to untie the genomic and metabolic complexity governing extremophiles-M&R interactions. Therefore, genomic approaches involving amplicon and whole-genome sequencing, comparative genomics and *in silico* genome mining have been employed to target essential genes encoding regulatory proteins, promoters, metal-binding proteins, enzymes, and biomolecules involved in radionuclides (e.g., Makarova et al., 2001; Methé et al., 2003; Zivanovic et al., 2009) and metals (e.g., Nies, 2000; Osorio et al., 2008; Cárdenas et al., 2010; Guo et al., 2014; Kernan et al., 2017) detoxification in extremophilic bacteria (e.g., *Geobacter sulfurreducens*, *Acidithiobacillus* spp., *Deinococcus radiodurans*, *Sulfobacillus thermosulfidooxidans*) and archaea (e.g., *Thermococcus gammatolerans*, *Metallosphaera sedula*).

Despite the relevance of this knowledge, the study of M&R-constrained gene expression and regulation through transcriptomics assumes a pivotal role for bioremediation purposes. DNA microarrays applied to the As-resistant *Thiomonas* spp. evidenced genomic rearrangements and horizontal gene transfer (Arsène-Ploetze et al., 2010), which can be exploited to transfer M&R resistance. Other studies observed overexpression of metallochaperones and enzymes mediating metal-phosphate granules formation in acidophilic bacteria (*A. ferooxidans*) (Navarro et al., 2009) and (thermo)acidophilic archaea (*Sulfolobus metallicus, Metallosphaera sedula*) challenged with M&Rs (Orell et al., 2013; Wheaton et al., 2016).

Nevertheless, many transcripts keep with unassigned functions or activities. Proteomics, through 2-DE gel coupled with mass spectroscopy (e.g., LC–MS, MALDI-ToF, and ICPL) tools and protein libraries, is thereby elucidating the type and abundance of proteins synthesized under M&R stress (Desai et al., 2010). Among them, those associated with EPS and biofilm production, oxidative stress responses, regulation of RND-type Cus system and metal uptake/efflux pumps (Zivanovic et al., 2009; Almárcegui et al., 2014; Martínez-Bussenius et al., 2016) are hugely relevant.

Additionally, metabolomics enlightens the metabolic fingerprinting of M&R-induced responses on extremophiles (Mosier et al., 2013). A diverse toolbox of chromatographic (GC and HPLC) and spectroscopic (FTIR and NMR) techniques have hence been employed to harness the genetic, transcriptional and translational processes sustaining the cellular biomolecules/metabolite fluxes (i.e., fluxomics) (Hold et al., 2009; Desai et al., 2010) leading to M&R sequestration. *In silico* reconstruction of unknown metabolic networks relevant for M&R bioremediation based on sequenced genomes (Sun et al., 2009), robust bioinformatics and libraries, is becoming a powerful fine-tuning approach, though requiring a cautious analysis of metal-microbe interactions to prevent misinterpretations (Johnson et al., 2015). In this context, community-level meta-omics (metagenomics, metatranscriptomics, and metaproteomics) offer great avenues by avoiding extremophiles culturing hurdles, while uncovering pools of novel biomolecules/enzymes for M&R bioremediation without previous sequence data (Mohamed et al., 2013), and providing ecologically-relevant analysis of community shifts and functions (Cowan et al., 2015; Garris et al., 2016; Mirete et al., 2016) along the bioremediation processes. Multi-‘omics’ integration, either at individual or community levels, constitutes another robust approach to unravel new M&R-bioremediation agents or monitoring bio-devices. Indeed, Wilkins et al. (2011) used a proteogenomic analysis to develop a biomarker of *G. sulfurreducens* activity during uranium bioremediation.

### Engineering Extremophic Microfactories for M&R Bioremediation

The advent of DNA recombinant techniques for tailoring existent abilities or to create synthetic ones, in natural or newly designed extremophiles, is a doorway to boost M&R bioremediation.

#### Genetic/Metabolic Engineering of Whole-Microbes and Communities

The peculiar mechanisms of extremophiles can be customized through the insertion of genes or gene clusters, as to enhance their skills and robustness for M&R attenuation (Cárdenas et al., 2010). Metal-resistant (*Cupriavidus metallidurans*; Rojas et al., 2011), radiophilic (*D. radiodurans*; Brim et al., 2000) and thermoacidophilic (*D. geothermalis*; Brim et al., 2003) bacteria were transformed with genes/clusters encoding enzymes involved in individual- or multi-M&Rs reduction/oxidation. This is biotechnologically worthy given the mixture of M&Rs in mine/nuclear wastes. In order to enhance M&R bioprecipitation, *D. radiodurans* was indeed manipulated to express a Cd(II)-binding synthetic phytochelatin (*EC20*) and metallothionein (*smtA*) (Chaturvedi and Archana, 2014), and a periplasmic acid phosphatase (PhoN), which hydrolysed organic into inorganic phosphates to precipitate uranium (Appukuttan et al., 2006, 2011). However, some extremophiles are more resistant to transformation due to their robust protective mechanisms (Chen et al., 2011; Liu et al., 2011), hence forcing the construction of adapted vectors (Meng et al., 2013).

Recently, the innovative project NANOBINDERS (PTDC/AAG-REC/3004/2014) is enrolled in the creation of biogenic nanopolymers functionalized to bind M&Rs (the NANOBINDERS) from uranium mine effluents. These M&R-binding nanopolymers are self-assembled inside a host transformed with new constructs harboring nanopolymer- and metal-binding-peptides-encoding genes, originally obtained from microbes isolated in a uranium mine. Such functional nanobeads are a revolutionary option to engineered microbes, which *in situ* release still raises regulatory concerns, besides genetic instability and limited efficiency under real scenarios (de Lorenzo, 2009). Alternatively, manipulated extremophiles may house suicidal genes (Paul et al., 2005) activated upon undetectable M&R levels, for future *in situ* application.

Native microbial communities have been used to rehabilitate M&R-contaminated areas by natural attenuation (Shukla et al., 2017), which can be engineered through biostimulation (i.e., supplementation of compounds/substrates enhancing microbial proliferation and activities) and/or bioaugmentation measures (i.e., addition of microbes endowed with particular functional mechanisms). In mesocosms, Ñancucheo and Johnson (2011) reduced acid mine drainage production when consortia of metabolically-cooperating microalgae, acidophilic and/or acid-tolerant bacteria were added to promote iron- and/or sulfate-reduction of tailings. The biostimulation of *Geobacter* sp. activity by acetate enrichment had enhanced uranium reduction, thereby proving successful *in situ* bioremediation (Anderson et al., 2003). However, metabolic fluxes modeling (Hold et al., 2009) should be applied in the future to design novel extremophilic consortia with tailored co-activities for M&R clean-up.

#### Synthetic Biology

Synthetic biology (SynBio) moves beyond the simple transformation of genes or operons to the insertion of newly designed and constructed synthetic biological parts and circuits to create robust, profitable, programmable and customized microfactories (Martínez-Garcia and de Lorenzo, 2016). The genetic instability and limited performance of engineered microbes for M&R bioremediation, can be overcome by DNA *de novo* synthesis and genome editing tools [e.g., Clustered Regularly Interspaced Short Palindromic Repeats (CRISPRs)- and protein Cas; Mougiakos et al., 2016, 2017]. Hence, extremophiles can be engineered as novel platforms (or *chassis*) housing stable and multiple designed functions together with the transcriptional and translational machinery to be active from laboratory- to field-scale extreme scenarios (Adams, 2016). Likewise, Gerber et al. (2015) took advantage of *Deinococcus* genetic plasticity and robustness to construct a *chassis* towards different applications. *Deinococcus* spp. was also successfully engineered with metal-resistance genes, demonstrating their genetic flexibility for heterologous and co-expression of different metal-resistance determinants under extreme temperature and radiation (Brim et al., 2000, 2003). Thus, constructing extremophilic-microfactories *chassis* is an appealing new wave for enhanced M&R bioremediation, especially if universal genetic toolkits could be created (Adams, 2016).

The joint endeavor of SynBio and metabolic engineering of communities is indeed enabling the design and building of synthetic consortia (Shong et al., 2012) for M&R remediation. Developing synthetic consortia demands the engineering of cell–cell communication, i.e., quorum-sensing (QS) systems (Brenner et al., 2008). QS modulates behavioral, metabolic and structural dynamics in microbial communities through signaling molecules [e.g., acyl homoserine lactones family (AHL)] (Shong et al., 2012). QS is pivotal namely in EPS synthesis and biofilm formation (Farah et al., 2005; McDougald et al., 2012), as well as AHLs can mediate copper resistance in *A. ferrooxidans* (Wenbin et al., 2011). Thus, putting effort on the modulation or creation of new QS systems and signaling molecules biosynthesis can fine-tune the pool of relevant functions in extremophilic consortia for M&R removal (e.g., biomass production and EPS synthesis) (Brune and Bayer, 2012; Shong et al., 2012).

## Generating Richness From Bioremediated M&Rs

A discouraging issue in M&R bioremediation is the disposal of final wastes. Nevertheless, extremophilic microfactories can be successfully explored for M&Rs recycling and/or recovery after being remediated, thereby sustainably closing M&R bioremediation processes.

### Biomining

Biomining is usually conducted by extremophilic bacteria and archaea for the recovery of M&R from ores and mine/metal-rich/nuclear wastes, through biooxidation (minerals oxidation for metal release) and/or bioleaching (metal solubilization) (Brune and Bayer, 2012; Johnson, 2014). These bioprocesses take advantage of the anaerobic metabolism, redox pathways, intercellular communication, biofilm formation (Vera et al., 2013), and resistance to heat, acid and metals of (thermo)acidophiles consortia (e.g., *Leptospirillum* spp., *Acidithiobacillus* spp., *Sulfobacillus* spp., *Ferroplasma* spp., *Acidiplasma* spp.) (Orell et al., 2010, 2013). Insoluble metal sulfides are converted into soluble metal sulfates by iron- and sulfur-oxidizing extremophiles, thereby facilitating the recovery of economically-relevant metals (e.g., Cu, Fe, Au, Ni, and Zn) (Watling et al., 2015; Wheaton et al., 2015).

### Microbial Fuel Cells (MFCs)

Microbial fuel cells (MFCs) can be a valuable recovery method since it uses the catalytic activity of microbes to generate electrical energy from the oxidation of organic substrates (Mathuriya and Yakhmi, 2014). They are composed by an anode that captures electrons from substrate oxidation, a cathode and an intermediate cation-specific membrane. Since M&Rs have a high redox potential, they can be reduced and precipitated by receiving electrons from the cathode. *Geobacter sulfurreducens* biofilms possessing electron-conductive nanowires (pili) have been exploited for increased electricity generation into MFCs (Reguera et al., 2006). The generated electrons can be further used to reduce U(VI) into removable U(IV), hence synergizing *Geobacter* sp. skills for bioremediation-derived-waste recycling. An anoxic sludge containing *Geobacter* spp. recovered the noble metal silver, while generating electrical energy in a MFC (Ho et al., 2017). Other extremophiles have indeed been used in MFCs (Shrestha et al., 2018), especially towards metal recovery (Dopson et al., 2015).

### Recycling Through Nanoparticles (NPs) Production

Extremophiles biosynthesize economically-lucrative inorganic NPs through intracellular or extracellular transformation/preci-pitation of M&Rs (Ulloa et al., 2016), hence providing another profitable M&R recycling strategy. The metal-tolerant *Cupriavidus metallidurans* CH34 mineralized Au and precipitated it as nanoparticulate Au^0^ in the periplasm (Reith et al., 2009), whilst the alkalotolerant *Rhodococcus* sp. accumulated Au-NPs in the cytoplasm and cell wall (Ahmad et al., 2003). Spherical Ag-NPs were also extracellularly synthesized at 80°C by the thermophilic *Ureibacillus thermosphaericus* (Juibari et al., 2011). CdS-NPs with enhanced stability under acidic conditions were produced by *Acidithiobacillus* spp., being a gainful option for Cd turnover (Ulloa et al., 2016). Uraninite, which is a worthy biogenic NP for uranium *in situ* remediation (Bargar et al., 2008), was synthesized by *Geobacter metallireducens* upon extracellular U(VI) reduction (Gorby and Lovley, 1992). The precious metal Pd(II) used in many industrial sectors was precipitated in *G. sulfurreducens* biofilms as Pd(0)-NPs and easily extracted by centrifugation (Yates et al., 2013).

## Concluding Remarks

Extremophiles enclose a pool of genetic and metabolic opportunities that can be harnessed as microfactories for multiple M&R-bioremediation hallmarks. Nevertheless, the complexity of extremophiles’ cellular mechanisms, interspecies relationships and M&R interplay under real scenarios of co-occurring extreme conditions may rise some challenges. Multi-‘omics’ applied side-by-side with genetic engineering techniques are covering knowledge gaps, allowing gene expression and metabolic pathways customization for improved bioremediation. Synthetic biology, however, through an iterative design-build-test-analyze framework is further revolutionizing M&R bioremediation and opening new perspectives on the creation of robust extremophilic recombinants or *chassis* with *de novo*-designed biologically-, energetically-, and economically-viable traits for specific M&R removal processes. Future trends will hence target adaptation or construction of multi-skilled extremophilic microfactories to sustainably empower M&R bioremediation and end-waste recycling, while keeping up with global changes, natural resources availability, and cost-efficiency requirements.

## Author Contributions

CM was responsible for doing all the tasks concerning the development of the work: conceptualized the idea and goals of the mini-review, performed literature search and revision, and wrote the manuscript.

## Conflict of Interest Statement

The author declares that the research was conducted in the absence of any commercial or financial relationships that could be construed as a potential conflict of interest.
